# Mapping of Enzyme Kinetics on a Microfluidic Device

**DOI:** 10.1371/journal.pone.0153437

**Published:** 2016-04-15

**Authors:** Hoon Suk Rho, Alexander Thomas Hanke, Marcel Ottens, Han Gardeniers

**Affiliations:** 1 Mesoscale Chemical Systems Group, MESA+ Institute for Nanotechnology, University of Twente, Enschede, The Netherlands; 2 BioProcess Engineering group, Department of Biotechnology, Faculty of Applied Sciences, Delft University of Technology, Delft, The Netherlands; University of Illinois at Chicago, UNITED STATES

## Abstract

A microfluidic platform or “microfluidic mapper” is demonstrated, which in a single experiment performs 36 parallel biochemical reactions with 36 different combinations of two reagents in stepwise concentration gradients. The volume used in each individual reaction was 36 nl. With the microfluidic mapper, we obtained a 3D enzyme reaction plot of horseradish peroxidase (HRP) with Amplex Red (AR) and hydrogen peroxide (H_2_O_2_), for concentration ranges of 11.7 μM to 100.0 μM and 11.1 μM to 66.7 μM for AR and H_2_O_2_, respectively. This system and methodology could be used as a fast analytical tool to evaluate various chemical and biochemical reactions especially where two or more reagents interact with each other. The generation of dual concentration gradients in the present format has many advantages such as parallelization of reactions in a nanoliter-scale volume and the real-time monitoring of processes leading to quick concentration gradients. The microfluidic mapper could be applied to various problems in analytical chemistry such as revealing of binding kinetics, and optimization of reaction kinetics.

## Introduction

Enzymes, biological catalysts, play important roles in various biological processes such as food fermentation, bio-analysis, protein synthesis, and drug discovery [1,2]. Enzymes can be characterized by their catalytic effect on reaction kinetics [3]. The essential information to understand the mechanism of enzyme-catalyzed reactions is the rate of reaction accelerated by the enzyme under different conditions. However, in most industrial and pharmaceutical applications, the rate of the enzymatic reactions are not only dependent on one major parameter but also two or more factors that interact with each other and strongly influence the behavior of the target system [4]. For example, apoenzymes require a cofactor, cosubstrate, or coenzyme to be functionalized as holoenzymes to catalyze the conversion of a substrate [2,5]. Hence the dynamic interactions between various effective parameters according to their concentrations has important implications on the characterization and optimization of enzyme systems, or more generally of reaction networks in the fields of Systems Biology [6].

Practically, to create a single three dimensional (3D) response plot [7,8], dual concentration gradients of two different reagents are required. Nevertheless, generating dual concentration gradients is almost impracticable with conventional test tubes and pipettes because only one test condition can be manually handled at a given time. Microtiter plate readers coupled with robotic fluid delivery systems can provide accurate gradient profiles and also dual concentration gradients of two reagents to obtain a 3D response. However, the long experimentation time and elaborate handling of reagents that are required for these methods hamper the observation in quantitative and amalgamative behaviors of target molecules. Furthermore, the involved high sample consumption is challenging because large volume causes very high cost in the early discovery phases of enzymes and substrates.

With a strong demand to generate the desired combinations of mixtures in an extremely small volumes, several microfluidic devices have been developed by adapting networking of continuous flows [9–18], parallel reactors [19–22], and micro-droplets [23–26]. These miniaturized devices have shown many clear advantages by applying concentration gradients on a chip. These include the requirement of low sample consumption and also the integration of sample preparation steps such as metering, mixing, incubation, and optical detection. Flow-based microfluidic systems provide a flexible linear gradient and also fast changing of the gradient profile which is beneficial for the study of cell biology, for example, cell culture [13,14] and chemotaxis [16–18]. However, the continuous tracing of an event in an individual reaction, which is critical to determine the rate of reaction, remains challenging for kinetic studies. The devices made by multilayer soft lithography are especially efficient in performing multiple reactions with the large scale integration of reactors [19–21,27–31]. With these devices the incubation of target mixtures in a closed system without fluid flow allowed both continuous monitoring and long term observation of the reactions. Although the previous devices performed multiple reactions with a series of concentrations of a single reagent, rapid generation of dual concentration gradients of different reagents is a desirable advancement, helpful in achieving a bird’s-eye view on a complicated enzyme system and in revealing the reaction network and interaction of factors in a certain target system.

Here, we developed an integrated system that can create dual reagent concentration gradients on a microfluidic chip, which allows the subsequent construction of 3D response plots from the generated concentration gradients. The device has 36 parallel reactors to conduct 36 individual enzyme reactions with concentration gradients of two substrates at a constant concentration of an enzyme. Using the system, we performed 36 horseradish peroxidase (HRP) catalyzed reactions with dual gradients of Amplex Red (AR) and hydrogen peroxide (H_2_O_2_). With a single experiment we obtained a 3D kinetic plot that contains all the detailed information for understanding the kinetics of the target enzyme system.

## Materials and Methods

### Chip design

**Fig 1:**
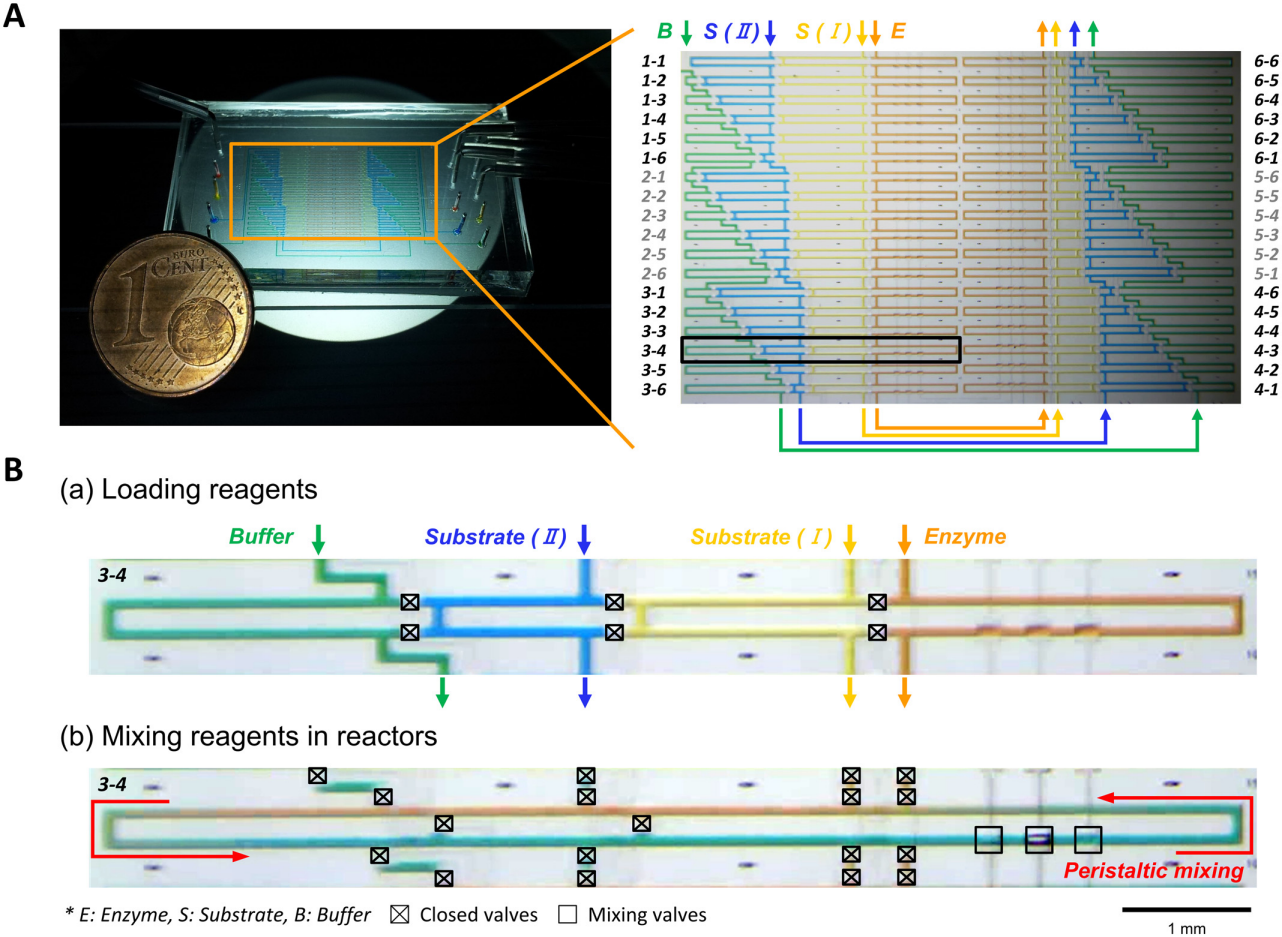
Design and operation of complete microfluidic device. (A) Photo of the device in connections with control ports (left) and 36 parallel reactors with food colored dyes loaded. (B) Operation of a single reactor element in the device. Metering valves were closed when the reagent solutions were loaded (a) and side valves were closed for the formation of a loop-shaped reactor (b). The solutions in the reactor were mixed by operating mixing valves in a sequence.

The microfluidic mapper consists of 36 parallel reactors as shown in Fig 1 (Photolithographic mask design is shown in S1 Fig). The fluidic channels were filled with food colored dyes to visualize the microchannel layout of the device. Fig 1A shows the connections of channels for loading four reagents, enzyme (orange color), substrate I (yellow color), substrate II (blue color), and buffer (green color) solutions. The mixing ratio of four reagents in a reactor is determined by the length of the channels. The final combinations and compositions of the reagents in the 36 reactors are summarized in Table 1. Fig 1B shows the process flow in a reactor. During loading reagents into the device, the reactor is separated into four loading sites for four reagents by closing metering valves. After loading, the metering valves are open while side valves are closed to form a single loop-shaped reactor. Then the mixing valves are actuated in a specific sequence, (100), (110), (010), (011), (001), (101), where 0 is open and 1 is closed, to generate fluid flow for active mixing of the reagents (S1 Movie).

**Table 1:** Combinations and compositions of reagents in 36 parallel reactors to accommodate dual concentration gradients of two substrates.

Reactor number	Channel length* [μm]				Volume [nl]				Final concentration		
	Enzyme	Substrate #1	Substrate #2	Buffer	Enzyme	Substrate #1	Substrate #2	Buffer	Enzyme (E_0_: initial Con.)	Substrate #1 (S1_0_: initial Con.)	Substrate #2 (S2_0_: initial Con.)
1–1	6000	6000	6000	0	12	12	12	0	0.333 E_0_	0.333 S1_0_	0.333 S2_0_
1–2	6000	6000	5000	1000	12	12	10	2	0.333 E_0_	0.333 S1_0_	0.278 S2_0_
1–3	6000	6000	4000	2000	12	12	8	4	0.333 E_0_	0.333 S1_0_	0.222 S2_0_
1–4	6000	6000	3000	3000	12	12	6	6	0.333 E_0_	0.333 S1_0_	0.167 S2_0_
1–5	6000	6000	2000	4000	12	12	4	8	0.333 E_0_	0.333 S1_0_	0.111 S2_0_
1–6	6000	6000	1000	5000	12	12	2	10	0.333 E_0_	0.333 S1_0_	0.056 S2_0_
2–1	6000	5000	6000	1000	12	10	12	2	0.333 E_0_	0.278 S1_0_	0.333 S2_0_
2–2	6000	6000	5000	2000	12	10	10	4	0.333 E_0_	0.278 S1_0_	0.278 S2_0_
2–3	6000	6000	4000	3000	12	10	8	6	0.333 E_0_	0.278 S1_0_	0.222 S2_0_
2–4	6000	6000	3000	4000	12	10	6	8	0.333 E_0_	0.278 S1_0_	0.167 S2_0_
2–5	6000	6000	2000	5000	12	10	4	10	0.333 E_0_	0.278 S1_0_	0.111 S2_0_
2–6	6000	6000	1000	6000	12	10	2	12	0.333 E_0_	0.278 S1_0_	0.056 S2_0_
3–1	6000	4000	6000	2000	12	8	12	4	0.333 E_0_	0.222 S1_0_	0.333 S2_0_
3–2	6000	6000	5000	3000	12	8	10	6	0.333 E_0_	0.222 S1_0_	0.278 S2_0_
3–3	6000	6000	4000	4000	12	8	8	8	0.333 E_0_	0.222 S1_0_	0.222 S2_0_
3–4	6000	6000	3000	5000	12	8	6	10	0.333 E_0_	0.222 S1_0_	0.167 S2_0_
3–5	6000	6000	2000	6000	12	8	4	12	0.333 E_0_	0.222 S1_0_	0.111 S2_0_
3–6	6000	6000	1000	7000	12	8	2	14	0.333 E_0_	0.222 S1_0_	0.056 S2_0_
4–1	6000	3000	6000	3000	12	6	12	6	0.333 E_0_	0.167 S1_0_	0.333 S2_0_
4–2	6000	6000	5000	4000	12	6	10	8	0.333 E_0_	0.167 S1_0_	0.278 S2_0_
4–3	6000	6000	4000	5000	12	6	8	10	0.333 E_0_	0.167 S1_0_	0.222 S2_0_
4–4	6000	3000	3000	6000	12	6	6	12	0.333 E_0_	0.167 S1_0_	0.167 S2_0_
4–5	6000	6000	2000	7000	12	6	4	14	0.333 E_0_	0.167 S1_0_	0.111 S2_0_
4–6	6000	6000	1000	8000	12	6	2	16	0.333 E_0_	0.167 S1_0_	0.056 S2_0_
5–1	6000	3000	6000	4000	12	4	12	8	0.333 E_0_	0.111 S1_0_	0.333 S2_0_
5–2	6000	6000	5000	5000	12	4	10	10	0.333 E_0_	0.111 S1_0_	0.278 S2_0_
5–3	6000	6000	4000	6000	12	4	8	12	0.333 E_0_	0.111 S1_0_	0.222 S2_0_
5–4	6000	3000	3000	7000	12	4	6	14	0.333 E_0_	0.111 S1_0_	0.167 S2_0_
5–5	6000	6000	2000	8000	12	4	4	16	0.333 E_0_	0.111 S1_0_	0.111 S2_0_
5–6	6000	6000	1000	9000	12	4	2	18	0.333 E_0_	0.111 S1_0_	0.056 S2_0_
6–1	6000	3000	6000	5000	12	2	12	10	0.333 E_0_	0.056 S1_0_	0.333 S2_0_
6–2	6000	6000	5000	6000	12	2	10	12	0.333 E_0_	0.056 S1_0_	0.278 S2_0_
6–3	6000	6000	4000	7000	12	2	8	14	0.333 E_0_	0.056 S1_0_	0.222 S2_0_
6–4	6000	3000	3000	8000	12	2	6	16	0.333 E_0_	0.056 S1_0_	0.167 S2_0_
6–5	6000	6000	2000	9000	12	2	4	18	0.333 E_0_	0.056 S1_0_	0.111 S2_0_
6–6	6000	6000	1000	10000	12	2	2	20	0.333 E_0_	0.056 S1_0_	0.056 S2_0_

* Channel width: 100 μm, channel height: 19.8 ± 0.3 μm (measured before reflowing process, n = 10)

### Chip fabrication

The device consists of two polydimethylsiloxane (PDMS) layers, a fluidic layer and a control layer, and was fabricated by multilayer soft lithography [32,33]. Masks were designed by CleWin software (WieWeb software, Hengelo, The Netherlands) and printed on 5” soda lime glasses by LBPG Heidelberg DWL200 (Heidelberg Instruments Mikrotechnik GmbH, Germany). Positive photoresist (AZ 40 XT, MicroChemicals GmbH, Ulm, Germany) was spun onto 4" silicon wafers. The wafers were exposed to UV light through masks and developed. The mold for the fluidic layer was reflowed by heating at 140°C for one minute. The fluidic layer was made by pouring uncured PDMS (GE RTV615, elastomer:cross-linker = 7:1) onto the mold. The control layer was produced by spin-coating uncured PDMS (elastomer:cross-linker = 20:1) onto the master mold at 2300 rpm for one minute. The fluidic layer was cured for 45 minutes at 80°C and peeled off from the mold. After punching holes for inlets and outlets with a 25-gauge punch (Syneo Co., Angleton, TX, USA) the fluidic layer was aligned over the control layer. The aligned layers were baked for 1 hour at 80°C, after which the layers were peeled off from the mold and holes for the connections with control ports were punched. The PDMS device was placed on a pre-cleaned glass slide (Fisher Scientific, Landsmeer, The Netherlands) and baked in the oven at 80°C for 12 hours to advance adhesion. To prevent non-specific binding of biomolecules, fluidic channels were treated with copolymer pluronic 10 g/L (Millipore, Zug, Switzerland) for 5 minutes before use and washed with reaction buffer solution for 30 minutes.

### Chip operation

The microfluidic device was controlled by a pneumatic control system. Micro-valves were operated by applying compressed nitrogen gas into control channels. The pneumatic control system was automated by combining pressure regulators, 3/2-way solenoid valves, and EasyPort USB digital I/O controller (all from Festo, Festo BV, The Netherlands). The system was automatically controlled by a custom-built LabVIEW (National Instruments Co.) program.

### Data processing

We used a stereo microscope (Motic SMZ168, Lab Agency Benelux BV, The Netherlands) equipped with a CMOS camera (Moticam 3.0) for recording peristaltic mixing phenomena in the device. We used an inverted fluorescent microscope (Leica DMI 5000M, 10X and 20X Objectives, Leica Microsystems BV, The Netherlands) equipped with an automatic XY-stage (Oasis PCI XY control unit), and a digital camera (Leica DFC300 FX, Leica Microsystems BV, The Netherlands) for acquisition of time-lapse images to monitor fluorescent intensities in each of the 36 reactors. Time interval between two acquired fluorescent images of neighboring reactors was 3 seconds and between two time-lapse images of the same reactor was 100 seconds (S2 Fig). All the acquired images were processed and analyzed by the time series analyzer of Image J software (http://rsb.info.nih.gov/ij/). Kinetic data were analyzed using a nonlinear regression analysis program (Enzyme Kinetic Module, SigmaPlot, Systat Software, Inc.).

### Chip validation

100 μM of fluorescein (Sigma-Aldrich Chemie BV, Zwijndrecht, The Netherlands) solution was prepared in Milli-Q water (Millipore Co.). The fluorescein solution was loaded into one loading site of the 36 reactors while the other three loading sites were filled with Milli-Q water. After mixing the solutions in the reactors for 1 minute, the fluorescent images of the 36 reactors were acquired by a Leica I3 filter cube (excitation: BP 450–490 nm; emission: LP 515 nm).

### Enzyme reaction

100 μM of resorufin (Sigma-Aldrich Chemie BV, Zwijndrecht, The Netherlands) solution was prepared in Milli-Q water for the generation of a standard curve. Amplex Red Hydrogen Peroxide/Peroxidase Assay Kit was obtained from Invitrogen (Invitrogen, Fisher Scientific, Landsmeer, The Netherlands). Stock solutions of 100 U/mL of HRP and 2 mM of AR were prepared in reaction buffer (0.25 M sodium phosphate, pH 7.4) and stored at -20°C. The stock solutions were diluted with reaction buffer immediately before use. 30% of hydrogen peroxide solution was obtained from Sigma-Aldrich (Zwijndrecht, The Netherlands) and diluted with Milli-Q water before use. The fluorescent signal from resorufin was observed by a Leica N 2.1 filter cube (excitation: BP 515–560 nm; emission: LP 590 nm). For the off-chip measurement of the resorufin fluorescent signal we used a spectrometer (Maya 200 Pro, Ocean Optics BV, Duiven, The Netherlands) with a xenon light source (HPX-2000, Ocean Optics BV, Duiven, The Netherlands) and a cuvette (fluorescence cell, 105.251-QS, Hellma BV, Nieuwegein, The Netherlands).

## Results and Discussion

### Device calibration (peristaltic mixing and metering)

**Fig 2:**
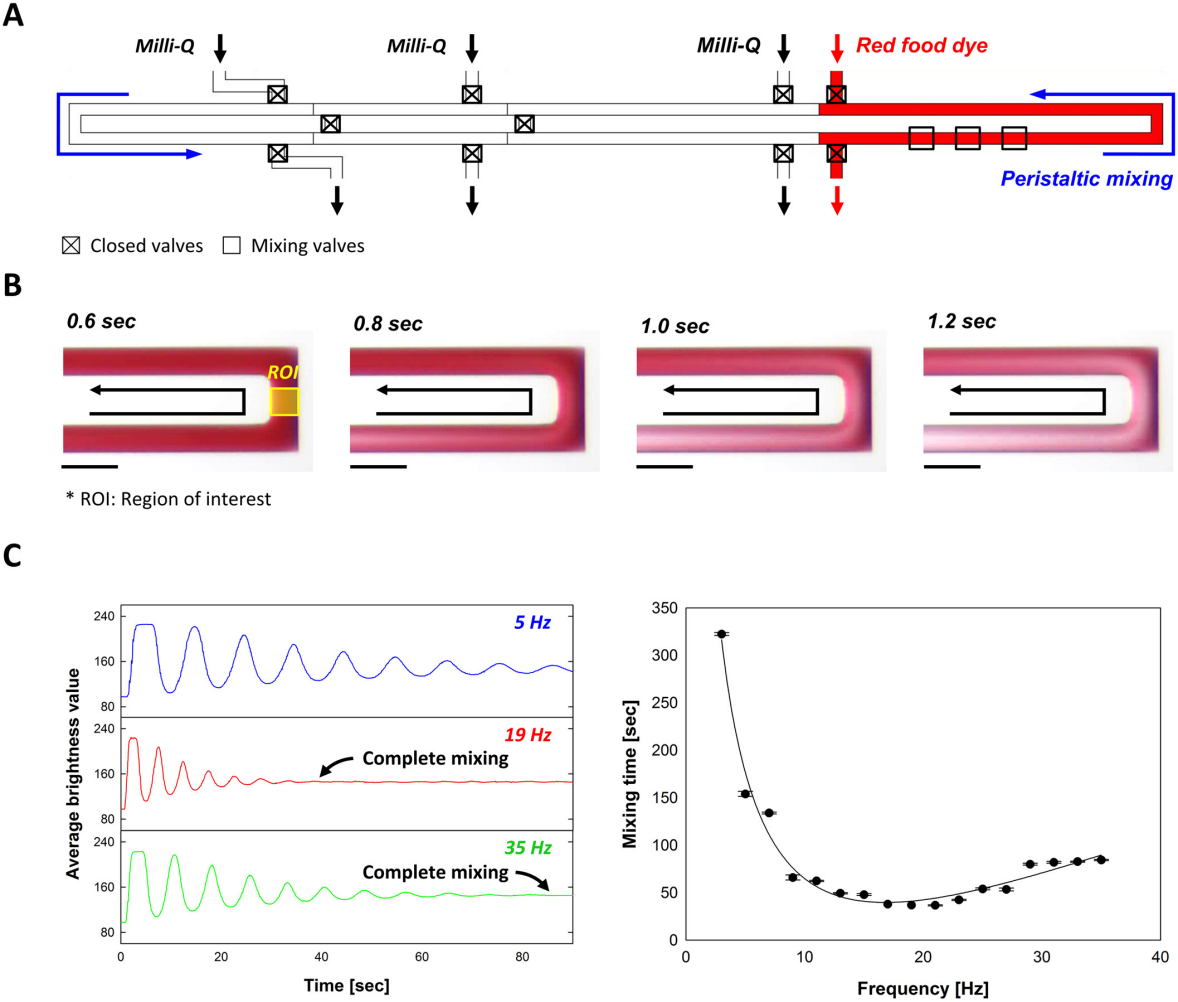
Peristaltic mixing on the device. (A) Composition of solutions for the study of mixing efficiency of the device, (B) Time-lapse microscope images of mixing of two liquid phases (200 μm scale bars are shown), and (C) Time series analysis of the change of average brightness value in a micro-mixer and relationship between the operating frequencies and complete mixing time.

Enhanced mixing and accurate metering are the most important capabilities of the device for the study of kinetics with parallel reactors. To validate and calibrate the device we tested the efficiency of our peristaltic mixing system and the accuracy in the generation of concentration gradient on the device. For the study of the mixing performance of the device we introduced red food dye solution into the loading site for an enzyme and Milli-Q water into the substrate I, substrate II, and buffer loading sites (Fig 2A). The three mixing valves were operated by cycling through the following six states (1 0 0), (1 1 0), (0 1 0), (0 1 1), (0 0 1), and (1 0 1), in sequence to achieve peristaltic mixing of the solutions in a reactor. The operating frequencies varied from 1 Hz to 35 Hz. Fig 2B shows time-lapse microscope images of mixing red dye solution and water. The circulation motion of the fluids was monitored and a time series analysis of the change of the average brightness values was performed at the ROI (region of interest, in Fig 2B) by Image J software. By operating the mixing valves in a sequence, Poiseuille flow was generated and the parabolic profile of the flow was stretched in the channel. The stretched interface between two liquid phases caused fast diffusion of the molecules. The mixing speed is dependent on the operating frequency of the valves. Fig 2C (left) shows the change of average brightness value at the ROI over time for various cycling frequencies. Corresponding to the degree of mixing, the fluctuation of the average brightness value steadily decreased until the average brightness reached a constant value. The complete mixing time, when the two solutions started to distribute uniformly, was evaluated to determine the mixing efficiency. Fig 2C (right) shows the relationship between the pumping frequencies and the complete mixing time. The mixing time is exponentially decreased with the increase of the frequency of the operating cycles below the cutoff frequency, 19 Hz [34]. The maximum operating frequency is approximately 35 Hz and matches with the limitation of the response time of the solenoid valves.

**Fig 3:**
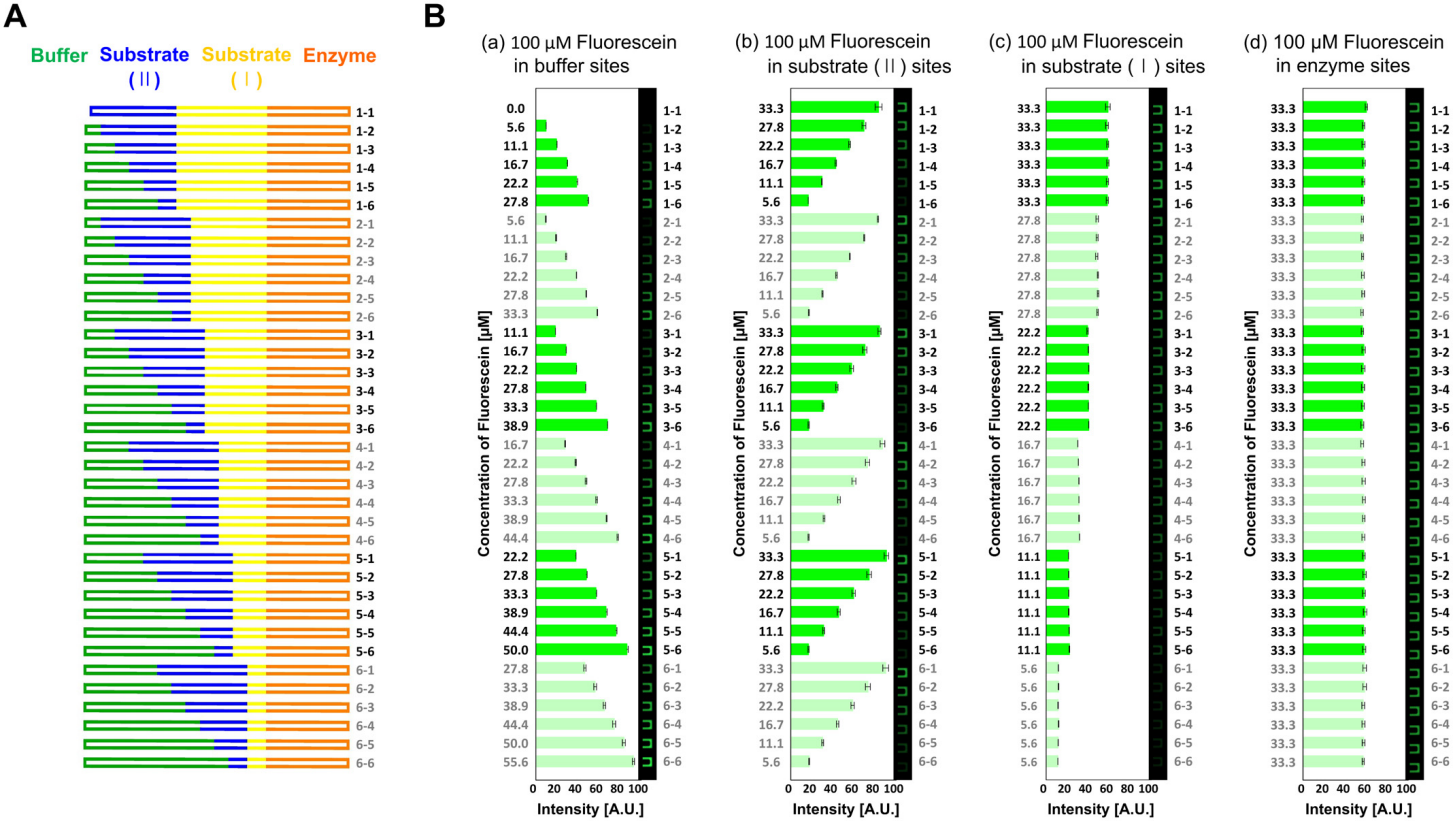
Generation of concentration gradient of fluorescein. (A) Design of the compositions of solutions in 36 reactors. Each reactor consists of four loading sites for enzyme (orange), substrate I (yellow), substrate II (blue), and buffer (green). (B) Fluorescence intensities of 36 reactors with concentration gradients of fluorescein as a negative control. The gradient of concentration of fluorescein was obtained by loading fluorescein solution into only one loading site while Milli-Q water was introduced into the other three sites. The obtained fluorescence intensities measured in the 36 reactors match with the target compositions (error bars represent the standard deviation from three measurements).

To demonstrate and validate the metering functionality of the device, we formed the concentration gradient of fluorescein and obtained fluorescence intensities in 36 reactors. Fig 3A shows the design of the compositions of reagents in the reactors. Each reactor has four loading sites for enzyme (orange), substrate I (yellow), substrate II (blue), and buffer (green) and the volumes of the reagents in the reactor are determined by the lengths of channels. As a negative control to validate the capability of the device on metering reagents we transferred 100 μM of fluorescein and Milli-Q water into the device. We filled one of the four loading sites with fluorescein solution and the remaining sites with Milli-Q water. Then we mixed the solutions for 1 minute by peristaltic mixing and obtained the fluorescent intensities of 36 reactors. Fig 3B shows the obtained fluorescent intensities of 36 reactors when only one loading site—(a) buffer, (b) substrate II, (c) substrate I, and (d) enzyme site—was filled with fluorescein solution and the other three sites were filled with Milli-Q. We performed each measurement three times and error bars in the graphs represent the standard deviation of the mean. The different fluorescent intensities correlated to the different metering ratios of the reagents in the reactors show accurate agreement with the target compositions of the reagents.

### Parallel enzymatic reactions on a chip

**Fig 4:**
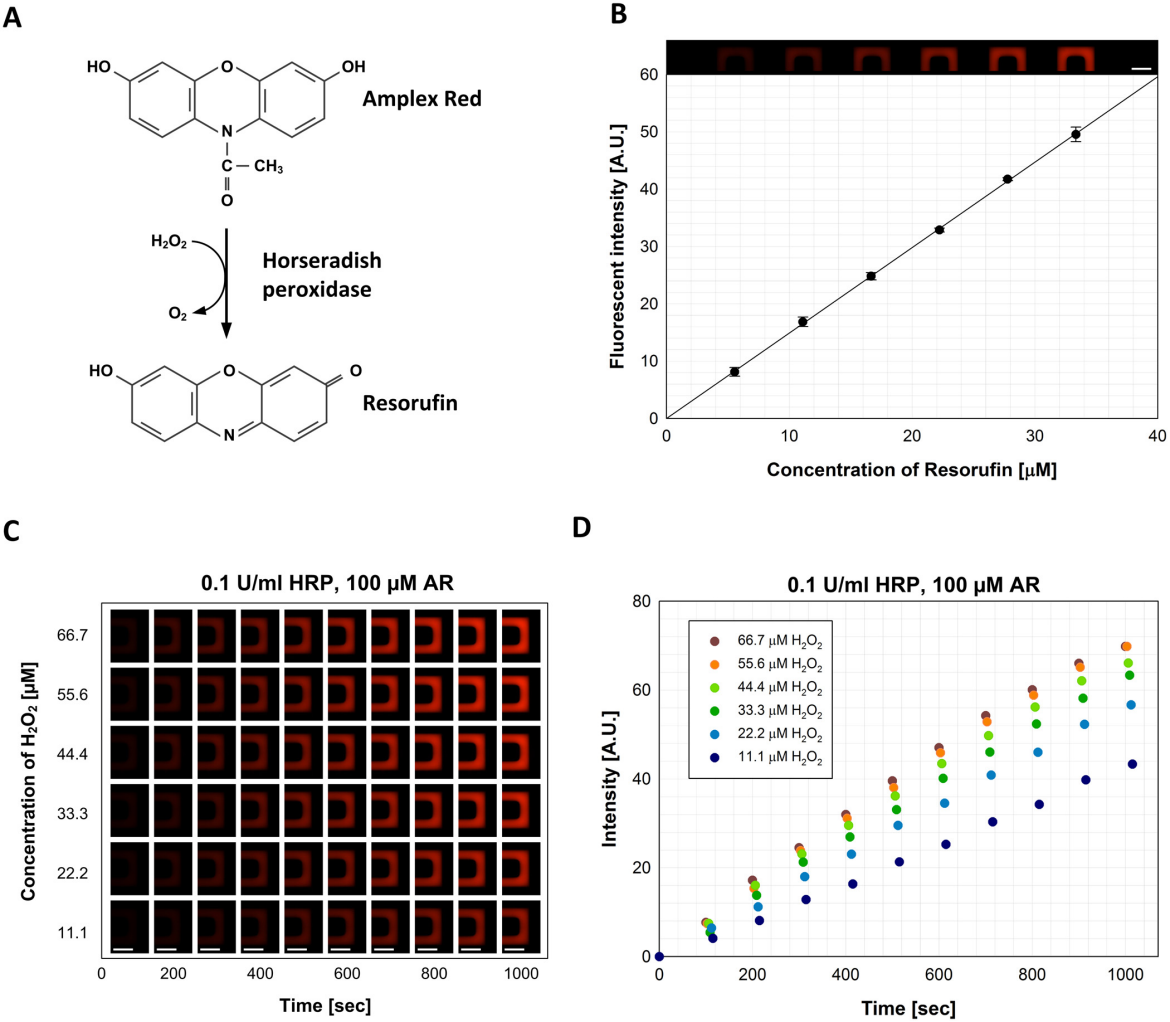
Parallel enzyme reactions on a chip. (**A)** Amplex Red, a nonfluorescent substrate, produces a highly fluorescent product, resorufin, in response to H_2_O_2_ upon the oxidation by HRP. (**B)** The relationship between the concentration of resorufin and its fluorescent intensity. The fluorescent images show the different concentrations of resorufin in reactors and the standard curve shows the change of fluorescent intensity with a response to the concentration of resorufin (200 μm scale bars are shown). (**C)** Time-lapse fluorescent images show resorufin formation in 6 reactors (reactor 1–1 to 1–6). (**D)** The increase in fluorescent intensity in the different reactors according to growing resorufin yield during enzymatic reactions of HRP with a constant concentration of AR and various concentrations of H_2_O_2_.

To demonstrate the feasibility of the device for a study of enzyme kinetics, we performed 36 parallel HRP-catalyzed reactions with a series of concentration changes of AR and H_2_O_2_. HRP oxidizes nonfluorescent AR to a highly fluorescent product, resorufin, by employing H_2_O_2_ as a cosubstrate (Fig 4A). [35] The kinetic properties of an enzyme are determined by the rate of reaction as a function of the concentrations of a substrate (S1 File). [2,3,36] Hence monitoring the resorufin yield oxidized by HRP according to the concentrations of two substrates, AR and H_2_O_2_, provides detailed information to understand the catalytic behavior of HRP as well as the attributions of the substrates in the reactions. Fig 4B shows a standard curve to quantify the concentration of resorufin according to its fluorescent intensity in the reactor for tracing the product formation in the reaction. The concentration gradient of resorufin ranging from 5.6 μM to 33.3 μM with an increment of 5.6 μM was obtained in the device by introducing 100 μM of resorufin into an enzyme loading site and reaction buffer into substrate I, substrate II, and buffer loading sites. The acquired fluorescent images show the different concentrations of resorufin in 6 reactors and the standard curve shows the fluorescent intensity according to the increase in the concentration of resorufin.

To determine the kinetic parameters, we measured the rates of 36 HRP-catalyzed reactions according to various concentrations of AR and H_2_O_2_ by filling the enzyme site with 0.3 U/ml of HRP, the substrate I site with 300 μM of AR, the substrate II site with 200 μM of H_2_O_2_, and buffer site with reaction buffer. Due to the pre-designed different metered volumes of reagents in each processor, the final concentration of HRP was 0.1 U/ml, and the final concentrations of AR and H_2_O_2_ were varied from 16.7 μM to 100.0 μM with an increment of 16.7 μM and ranged from 11.1 μM to 66.7 μM with an increment of 11.1 μM, respectively (S1 Table). After mixing the reagents time-lapse fluorescent images of the reactors were acquired for the kinetic traces. The time courses of the product yield in 6 reactors from 1–1 to 1–6 are shown in Fig 4C and 4D (additional data for all 36 reactors in S3 Fig). The increase in the fluorescent intensity was attributed to the formation of fluorescent product, resorufin. Since the concentrations of HRP and AR are constant, the different rates of reactions are only influenced by the H_2_O_2_ concentration in the 6 reactors.

**Fig 5:**
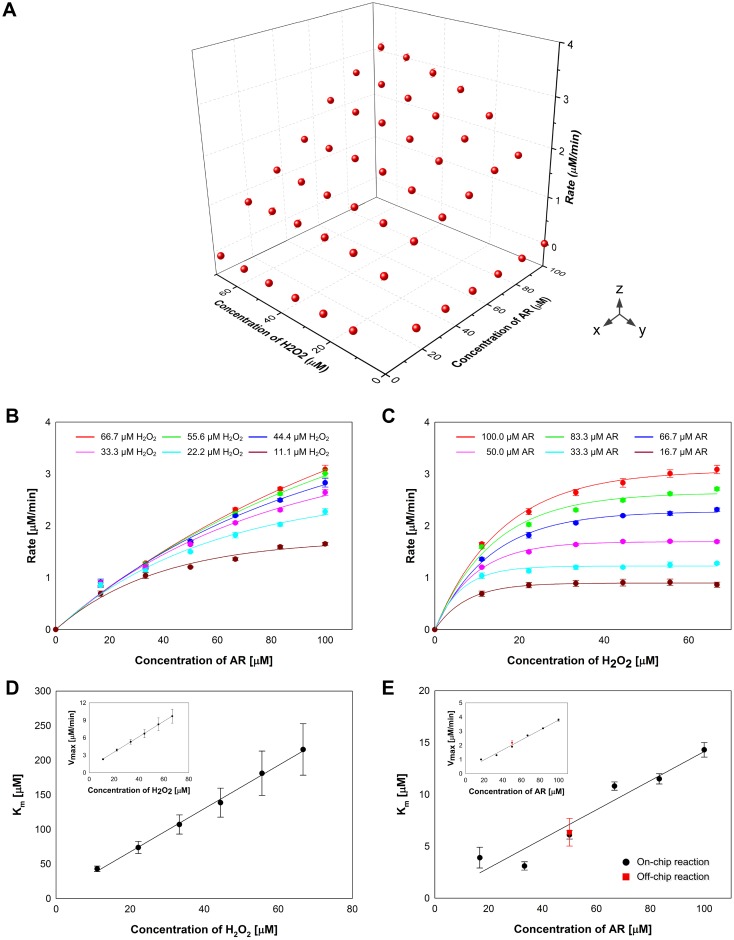
Michaelis-Menten plots of the enzymatic reaction of HRP with AR and H_2_O_2_. The initial rates of the HRP-catalyzed reactions with various concentrations of two substrates are plotted in 3D **(A)**. Based on xz projection **(B)** and yz projection **(C)** of the 3D plot, K_m_ and V_max_ values are calculated and plotted in **(D)** and **(E)**. The K_m_ and V_max_ values from off-chip reactions are shown in (E).

The initial rates of the reactions for 10 minutes after mixing were fitted with the Michaelis-Menten equation and kinetic parameters, K_m_ and V_max_, were determined by using a nonlinear regression analysis program, Sigmaplot Enzyme Kinetics Module. Fig 5A shows the rates of HRP-catalyzed reactions with dual concentration gradients of AR and H_2_O_2_ in a 3D plot, where x-axis, y-axis, and z-axis represent the AR concentration, the H_2_O_2_ concentration, and the rate of reaction, respectively. With the 3D plot detailed information about the influences of AR and H_2_O_2_ on the kinetics was obtained by plot projections on the xz and yz planes. Fig 5B shows the xz projection of the 3D plot. Based on a single Michaelis-Menten plot in the projection that shows the change of the reaction rate according to the concentration of AR at a specific concentration of H_2_O_2_, the kinetic parameters, K_m_ and V_max_, were calculated (S2 Table). In the same manner we determined K_m_ and V_max_ at 6 different concentrations of H_2_O_2_ and the relationship between K_m_ (or V_max_) and the H_2_O_2_ concentration was plotted in Fig 5D. The Michaelis-Menten plots obtained by the yz projection of the 3D plot and K_m_ and V_max_ determined by the Michaelis-Menten plots are shown in Fig 5C and 5E. We performed off-chip HRP-catalyzed reactions with a volume of 100 μl at various concentrations of H_2_O_2_, 2.5 μM, 5 μM, 10 μM, 15 μM, and 20 μM, and a constant concentration of AR, 50 μM (the detailed information is provided in S4 Fig). The values of K_m_ and V_max_ obtained from the off-chip experiments were 6.4 ± 1.3 and 2.2 ± 0.2, respectively (Fig 5E). Comparing with the values of K_m_ and V_max_ from the on-chip reactions, 6.1 ± 0.4 and 1.9 ± 0.0, the deviation was 5.3% for K_m_ and 13.7% for V_max_.

## Conclusions

Dual-gradient analysis and 3D reaction plots have been used by researchers for the fast evaluation of reaction characteristics [20,37,38]. Using microtiter-plates and pipettes, the dual-gradient methodology can produce a single 3D plot instead of two 2D plots. A wider spread of the concept in chemistry and biology has been limited mainly by issues of time, man power and material consumption. And despite the fact that 3D reaction profiling and 3D binding kinetics have been reported for proteomics and genomics, there are but a few tools to show 3D data profiling from a single experiment. Automated microfluidics, which works with nanoliter-scale reagents, can fulfil the need for such tools. For that to become possible, flexible protocols for the creation of concentration gradients in microfluidic devices is desired, because each application demands a different concentration range and a different number of reagents [21,22,39]. Commonly, flexibility can be achieved by adopting a modular approach, which is used frequently in engineering design. With our new device approach, we have achieved the desired flexibility in concentration gradient generation by using parallel mixer-reactor architectures. Based on the concentration of reagents loaded into the reactors, different concentrations can be achieved in an automated way, by controlled mixing and dilution. The device also includes different loading sites in each reactor to generate the concentrations of positive and negative controls for the characterization of reactions, in a single experiment. In addition, by using time-wise tracing of kinetic reactions and plotting them as a 3D landscape plot, characterization of kinetic reactions is possible with the resulting determination of kinetic parameters, such as K_m_ and V_max_ even for multiple substrate systems. This is one of the novel features of the present device. This flexibility in fast characterization of reactions could be expanded for various kinetic assays such as enzymatic kinetics, binding kinetics, and drug discovery.

The 3D reaction plot from a single experiment is useful for the fast evaluation of enzyme-catalyzed reactions. 3D plots could be constructed for single enzyme and two substrates system, single substrate with enzyme and coenzyme system, or single enzyme and single substrate with different pH [2,3]. If the final goal of the kinetic experiments is to evaluate the performance of reagents, then the rigor of mathematical analysis to determine kinetic parameters could be avoided, as rapid evaluation could be possible from the direct observation of 3D plot.

## Supporting Information

S1 Fig Photolithographic mask design (Clewin software) of a microfluidic mapper. (TIF)

S2 Fig Image acquisition of 36 reactors. Time interval between two acquisitions of the fluorescent images of neighboring reactors (ΔT) was 3 seconds and between two time-lapse images of the same reactor (T_n+1_ –T_n_) was 100 seconds for the kinetic study in this work.(A) The moving direction of an automatic stage for scanning 36 reactors, and (B) An example of two time-lapse image acquisitions and plotting of the intensity according to time for 6 reactors.
(TIF)

S3 Fig Time-lapse fluorescent images and time courses of HRP reactions. (A) Reactor 1–1 to 1–6, (B) Reactor 2–1 to 2–6, (C) Reactor 3–1 to 3–6, (D) Reactor 4–1 to 4–6, (E) Reactor 5–1 to 5–6, and (F) Reactor 6–1 to 6–6 (200 μm scale bars are shown).
(TIF)

S4 Fig Off-chip HRP-catalyzed reactions. (A) Standard curve to quantify the concentration of resorufin according to its fluorescence intensity for tracing the product formation in the HRP-catalyzed reaction. The fluorescence intensity of resorufin was measured at various concentrations of resorufin ranged from 10 μM to 60 μM with an increment of 10 μM (n = 3). (B) The change of fluorescence intensity of resorufin according to resorufin yield during HRP reactions. The increases in the resorufin fluorescence intensities were monitored at various concentrations of H_2_O_2_ (0.1 U/ml of HRP and 50 μM of AR). (C) Michaelis-Menten plot of the reactions. The initial rates of the reactions for 10 minutes were fitted with the Michaelis-Menten equation and kinetic parameters were calculated by Sigmaplot Enzyme Kinetic Module. The obtained K_m_ and V_max_ were 6.4 ± 1.3 and 2.2 ± 0.2, respectively (n = 3) Comparing with the values of K_m_ and V_max_ from the on-chip reactions, 6.1 ± 0.4 and 1.9 ± 0.0, the deviation was 5.3% for K_m_ and 13.7% for V_max_.
(TIF)

S1 File Michaelis-Menten equation. (PDF)

S1 Movie Operation of a microfluidic mapper. (AVI)

S1 Table Combinations and compositions of reagents for HRP reactions with AR and H_2_O_2_. (PDF)

S2 Table Michaelis-Menten reaction parameters for AR and H_2_O_2_ in the various conditions. (PDF)
