# Supplementary figures and images for: Mapping of Enzyme Kinetics on a Microfluidic Device
Source: PLoS One. 2016 Apr 15;11(4):e0153437. doi: 10.1371/journal.pone.0153437 (PMC4833427; doi:10.1371/journal.pone.0153437)

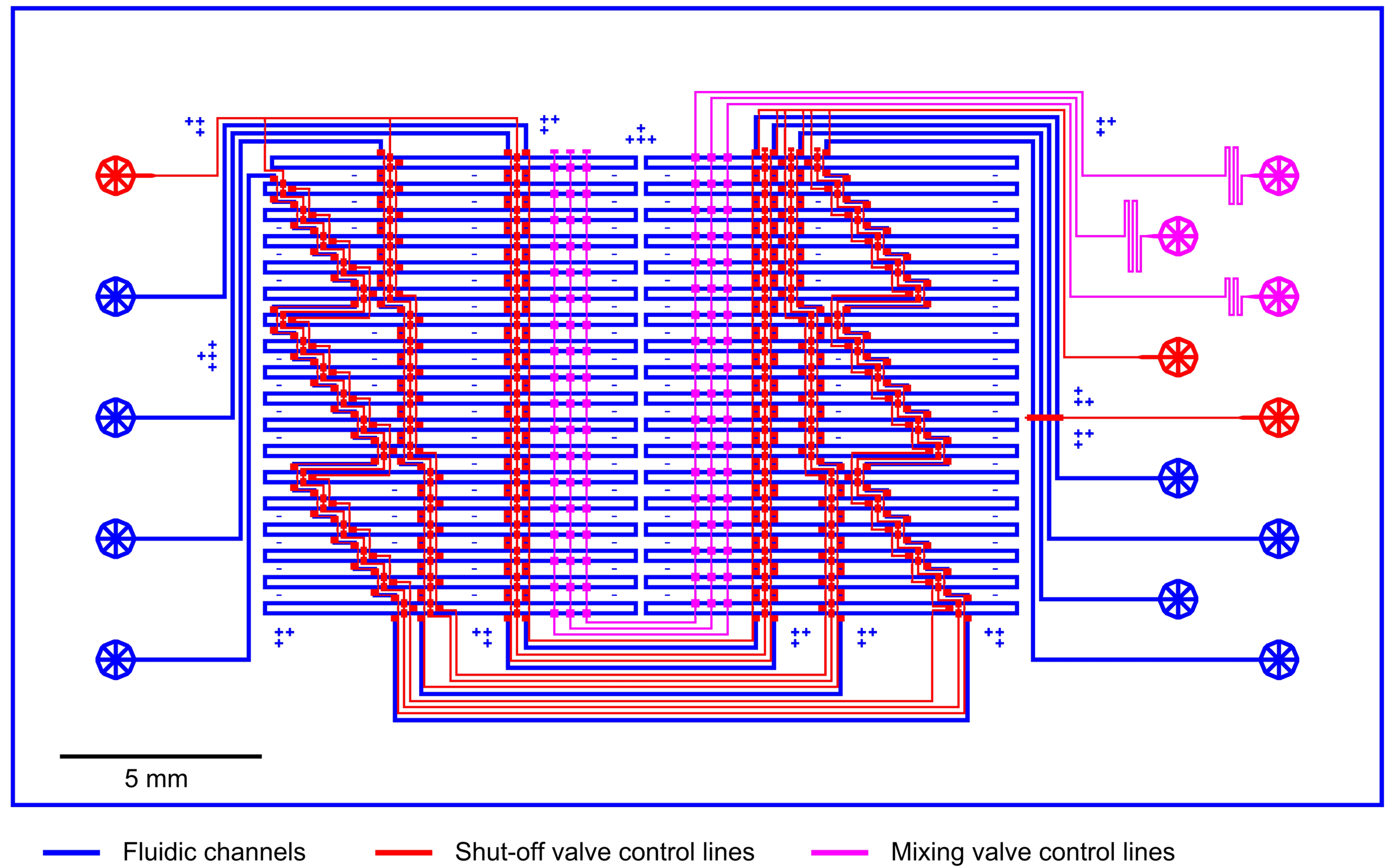

Supplement: S1 Fig — (TIF) [file pone.0153437.s001.tif]

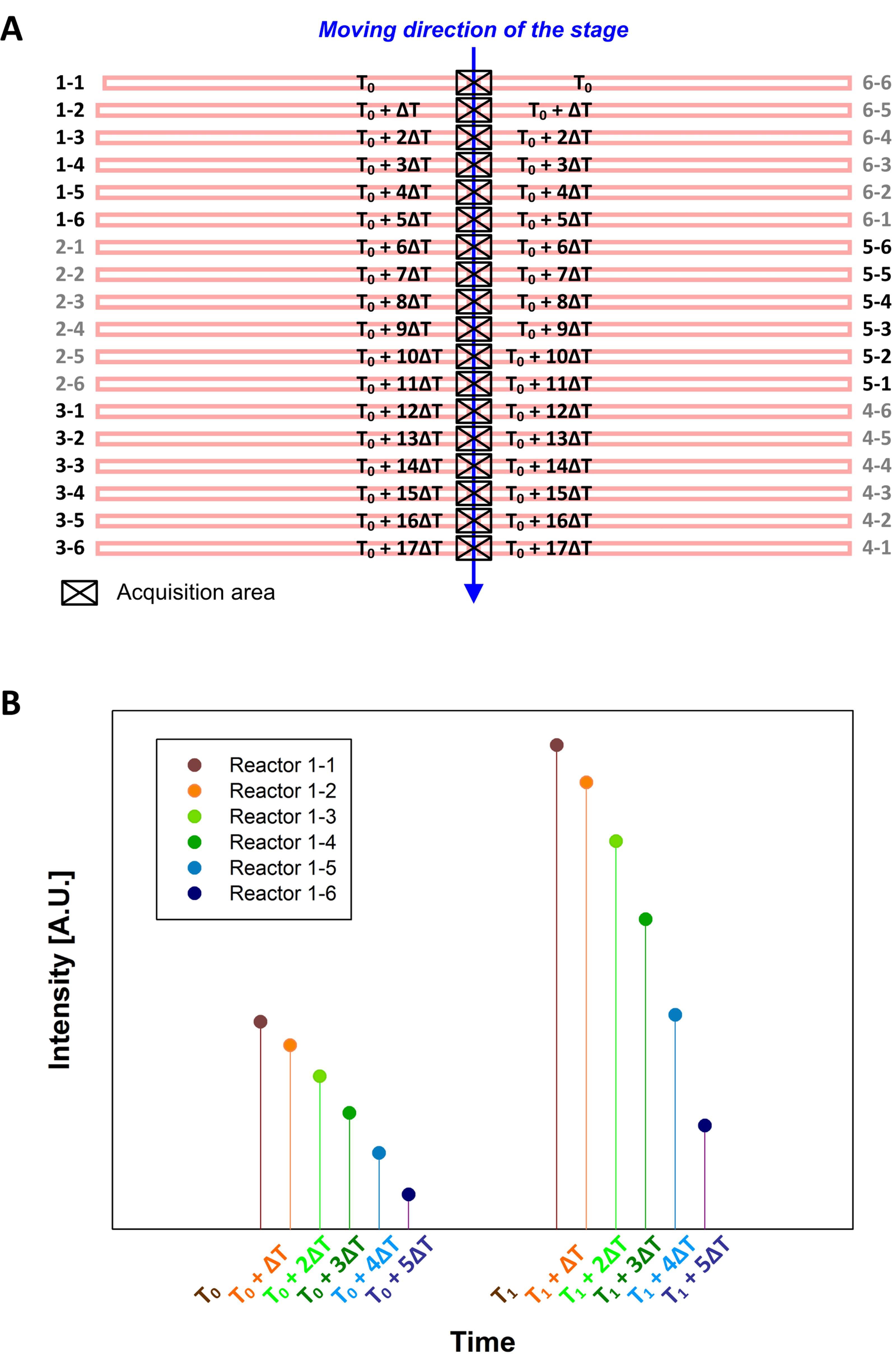

Supplement: S2 Fig — Time interval between two acquisitions of the fluorescent images of neighboring reactors (ΔT) was 3 seconds and between two time-lapse images of the same reactor (Tn+1 –Tn) was 100 seconds for the kinetic study in this work.(A) The moving direction of an automatic stage for scanning 36 reactors, and (B) An example of two time-lapse image acquisitions and plotting of the intensity according to time for 6 reactors. (TIF) [file pone.0153437.s002.tif]

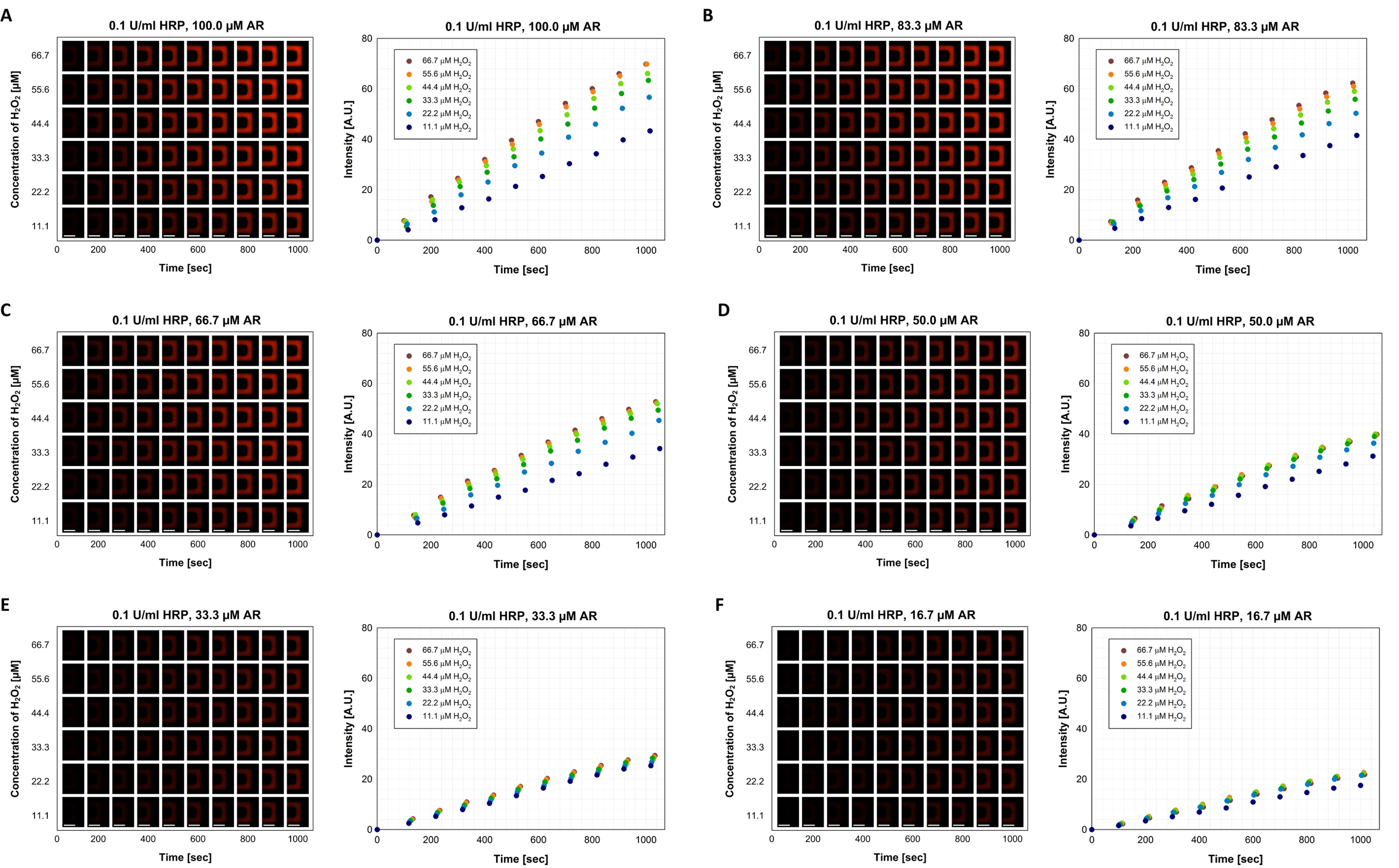

Supplement: S3 Fig — (A) Reactor 1–1 to 1–6, (B) Reactor 2–1 to 2–6, (C) Reactor 3–1 to 3–6, (D) Reactor 4–1 to 4–6, (E) Reactor 5–1 to 5–6, and (F) Reactor 6–1 to 6–6 (200 μm scale bars are shown). (TIF) [file pone.0153437.s003.tif]

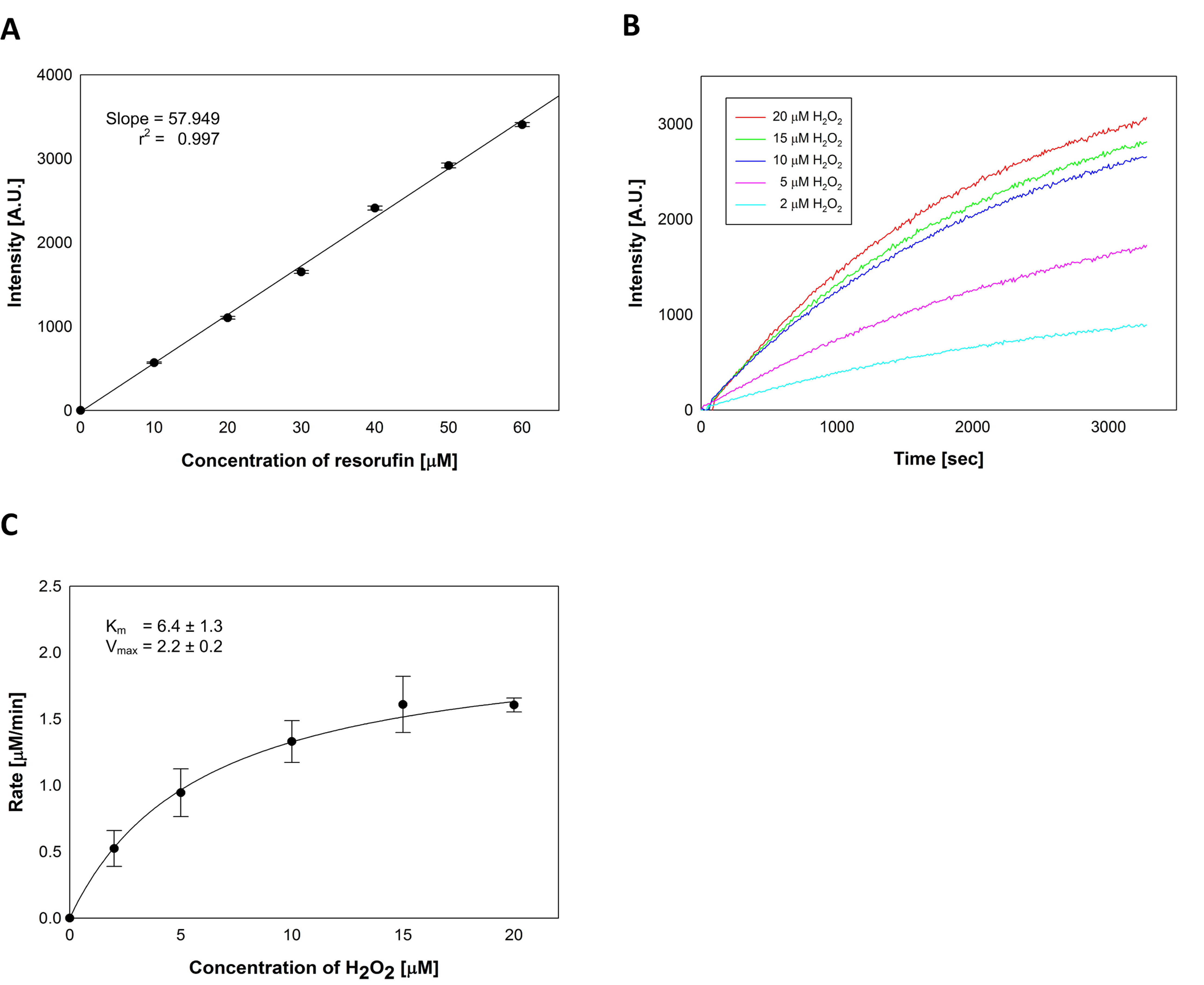

Supplement: S4 Fig — (A) Standard curve to quantify the concentration of resorufin according to its fluorescence intensity for tracing the product formation in the HRP-catalyzed reaction. The fluorescence intensity of resorufin was measured at various concentrations of resorufin ranged from 10 μM to 60 μM with an increment of 10 μM (n = 3). (B) The change of fluorescence intensity of resorufin according to resorufin yield during HRP reactions. The increases in the resorufin fluorescence intensities were monitored at various concentrations of H2O2 (0.1 U/ml of HRP and 50 μM of AR). (C) Michaelis-Menten plot of the reactions. The initial rates of the reactions for 10 minutes were fitted with the Michaelis-Menten equation and kinetic parameters were calculated by Sigmaplot Enzyme Kinetic Module. The obtained Km and Vmax were 6.4 ± 1.3 and 2.2 ± 0.2, respectively (n = 3) Comparing with the values of Km and Vmax from the on-chip reactions, 6.1 ± 0.4 and 1.9 ± 0.0, the deviation was 5.3% for Km and 13.7% for Vmax. (TIF) [file pone.0153437.s004.tif]
