# Supplementary material for: Mapping of Enzyme Kinetics on a Microfluidic Device
Source: PLoS One. 2016 Apr 15;11(4):e0153437. doi: 10.1371/journal.pone.0153437 (PMC4833427; doi:10.1371/journal.pone.0153437)
Supplement: S1 File — (PDF) [file pone.0153437.s005.pdf]

S1 File. Michaelis-Menten equation [1-3].

The kinetics of enzymes is generally governed by the Michaelis-Menten equation as follows:

$$V_0 = \frac{V_{max}\,[S]}{(K_m + [S])}$$

, where V_0, [*S*], V_{max}, and K_m represent the initial rate of product generation, the concentration of a substrate, maximum rate, and Michaelis-Menten constant, respectively. The initial rate increases with increasing [*S*], asymptotically approaching the maximum rate of an enzyme mediated reaction V_{max}. K_m is defined as the substrate concentration at which the initial rate reaches half of V_{max}.

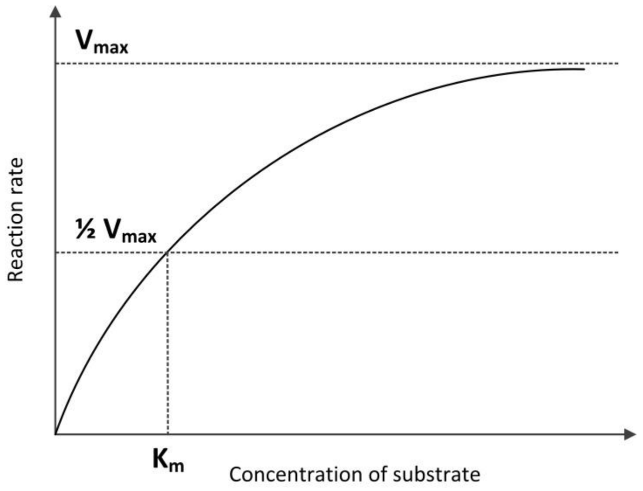

References

1. Lineweaver, H. and D. Burk, *The determination of enzyme dissociation constants.* Journal of the American Chemical Society, 1934. **56**(3): p. 658-666.
2. Segel, I.H., *Enzyme kinetics*. Vol. 360. 1975: Wiley, New York.
3. Shuler, M.L. and F. Kargi, *Bioprocess engineering*. 2002: Prentice Hall New York.
