# Supplementary material for: Mapping of Enzyme Kinetics on a Microfluidic Device
Source: PLoS One. 2016 Apr 15;11(4):e0153437. doi: 10.1371/journal.pone.0153437 (PMC4833427; doi:10.1371/journal.pone.0153437)
Supplement: S1 Table — (PDF) [file pone.0153437.s007.pdf]

S1 Table. Combinations and compositions of reagents for HRP reactions with AR and H_2O_2.

Reactor number	HRP [U/ml]	Amplex Red [μM]	H_2O_2 [μM]
1-1	0.1	100.0	66.7
1-2	0.1	100.0	55.6
1-3	0.1	100.0	44.4
1-4	0.1	100.0	33.3
1-5	0.1	100.0	22.2
1-6	0.1	100.0	11.1
2-1	0.1	83.3	66.7
2-2	0.1	83.3	55.6
2-3	0.1	83.3	44.4
2-4	0.1	83.3	33.3
2-5	0.1	83.3	22.2
2-6	0.1	83.3	11.1
3-1	0.1	66.7	66.7
3-2	0.1	66.7	55.6
3-3	0.1	66.7	44.4
3-4	0.1	66.7	33.3
3-5	0.1	66.7	22.2
3-6	0.1	66.7	11.1
4-1	0.1	50.0	66.7
4-2	0.1	50.0	55.6
4-3	0.1	50.0	44.4
4-4	0.1	50.0	33.3
4-5	0.1	50.0	22.2
4-6	0.1	50.0	11.1
5-1	0.1	33.3	66.7
5-2	0.1	33.3	55.6
5-3	0.1	33.3	44.4
5-4	0.1	33.3	33.3
5-5	0.1	33.3	22.2
5-6	0.1	33.3	11.1
6-1	0.1	16.7	66.7
6-2	0.1	16.7	55.6
6-3	0.1	16.7	44.4
6-4	0.1	16.7	33.3
6-5	0.1	16.7	22.2
6-6	0.1	16.7	11.1
