# Supplementary material for: Mapping of Enzyme Kinetics on a Microfluidic Device
Source: PLoS One. 2016 Apr 15;11(4):e0153437. doi: 10.1371/journal.pone.0153437 (PMC4833427; doi:10.1371/journal.pone.0153437)
Supplement: S2 Table — (PDF) [file pone.0153437.s008.pdf]

S2 Table. Michaelis-Menten reaction parameters for AR and H_2O_2 in the various conditions.

Amplex Red (AR)			Hydrogen peroxide (H_2O_2)		
Concentration of H_2O_2 [μM]	K_m [μM]	V_{max} [μM/min]	Concentration of AR [μM]	K_m [μM]	V_{max} [μM/min]
66.7	215.5 ± 37.2	9.7 ± 1.2	100.0	14.3 ± 0.7	3.8 ± 0.1
55.6	181.1 ± 32.1	8.3 ± 1.1	83.3	11.5 ± 0.5	3.2 ± 0.0
44.4	138.6 ± 21.0	6.7 ± 0.7	66.7	10.8 ± 0.4	2.7 ± 0.0
33.3	107.0 ± 13.9	5.3 ± 0.4	50.0	6.1 ± 0.4	1.9 ± 0.0
22.2	73.7 ± 8.8	3.9 ± 0.2	33.3	3.1 ± 0.4	1.3 ± 0.0
11.1	42.9 ± 3.9	2.3 ± 0.1	16.7	3.9 ± 1.0	1.0 ± 0.0
